# Utility of Authentic ^13^C‐Labeled Disaccharide to Calibrate Hyaluronan Content Measurements by LC‐MS

**DOI:** 10.1002/pgr2.70010

**Published:** 2024-11-05

**Authors:** Eduardo Stancanelli, Dixy E. Green, Katelyn Arnold, Jianxiang Zhang, Deyu Kong, Paul L. DeAngelis, Jian Liu

**Affiliations:** ^1^ Division of Chemical Biology and Medicinal Chemistry, Eshelman School of Pharmacy University of North Carolina Chapel Hill North Carolina USA; ^2^ Department of Biochemistry and Physiology The University of Oklahoma Health Science Center Oklahoma City Oklahoma USA

**Keywords:** 13C labeled disaccharide, chemoenzymatic synthesis, glycosaminoglycan, hyaluronic acid, LC‐MS/MS, quantification

## Abstract

Hyaluronan (hyaluronic acid, HA), a key glycosaminoglycan in the extracellular matrix, plays crucial roles in various physiological and pathological processes, including development, tissue hydration, inflammation, and tumor progression. Traditional methods for HA quantification, such as ELISA‐like assays, often have limitations in sensitivity and specificity, particularly for lower molecular weight HA. In this work, we introduce a coupled liquid chromatographic‐tandem mass spectrometric (LC‐MS/MS) method that employs a chemoenzymatically synthesized ^13^C‐labeled lyase‐derived authentic HA disaccharide calibrant for quantification of HA at the nanogram level. The method was validated against three HA polysaccharides with the sizes of ~33, 210, and 540 kDa. We applied this quantification technique to mouse tissues and plasma from both healthy and acetaminophen‐induced acute liver injury mice. Our data revealed a ~75‐fold increase in HA concentration in the liver of acetaminophen‐injured mice with a concomitant depletion from plasma. Overall, our method offers a robust, universal, and highly sensitive tool for HA analysis in diverse biological samples that will advance the investigation of the roles of this polysaccharide in human disease conditions.

**Abbreviations**
APAP: acetaminophen
ELISA: enzyme‐linked immunosorbent assay
GAG: glycosaminoglycan
HA: hyaluronic acid
HAS: hyaluronic acid synthase
HMW: high molecular weight
HSQC: heteronuclear single quantum coherence
LMW: low molecular weight
MW: molecular weight
NMR: nuclear magnetic resonance
*Pm*HAS: *Pasteurella multocida* hyaluronic acid synthase
UDP‐GlcNAc: uridine diphospho *N*‐acetyl glucosamine
UDP‐GlcA: uridine diphospho glucuronic acid

## 1 Introduction

Glycosaminoglycans (GAGs) are a group of complex macromolecules composed of repeating disaccharide units that are ubiquitously present in mammalian tissues and biofluids and play a pivotal role in the structural integrity and biochemical functions of the cells [1, 2, 3, 4]. Hyaluronic acid (hyaluronan or HA) stands out among GAGs for its simple structure and the fact that, unlike other GAGs, it is not built on a core protein and does not require additional modification such as sulfation of functional groups in the Golgi apparatus [5]. HA, solely composed of alternating repeat units of [d‐glucuronic acid β (1 → 4)*N‐*acetyl‐d‐glucosamine β (1 → 3)‐], is a key building block of the extracellular matrix in various vertebrate tissues [6, 7, 8]. HA is essential for lubricating synovial joints due to its ability to retain water [9]. HA also influences angiogenesis, which affects cancer development, and it is also involved in the immune response and regulation [10, 11].

Many of the HA polysaccharide's functions are strictly correlated with its molecular mass [12], which in healthy tissue is between approximately 1000–8000 kDa [13, 14]. Mammalian HA is synthesized by HA synthases (HASs), transmembrane enzymes, with three isoforms, namely, HAS1, 2, or 3 [15, 16]. It appears that the three HAS isoforms produce polymers with different average chain lengths [17]. In addition, the HASs generally produce high molecular weight HA (HMW > 500 kDa), while on the other hand, degradation of HMW HA by hyaluronidases and/or reactive oxygen species seem to be the main source of low molecular weight HA fragments (LMW < 500 kDa) [18, 19]. In recent years, altered HA metabolism and size has been linked to a variety of pathological conditions including rheumatic arthritis and osteoarthritis [20, 21], chronic hepatitis [22], lung metastatic cancer [23], and ovarian cancer.

Therefore, a sensitive and reliable method for measuring the levels of HA in biological samples gained substantial interest because several studies suggest a correlation between changes in HA content and pathological processes [24, 25, 26]. HA appears to regulate the microenvironment of tumors, often promoting the expression of malignant phenotype. High levels of HA have been identified in breast, colorectal, and prostate cancer, among others [27, 28, 29]. For most biological studies, enzyme‐linked sorbent assays (ELISA‐like assays) are the commonly used method for the determination of HA content [30, 31, 32]. Unlike ELISAs that typically employ an antibody‐based affinity reagent, the specificity for HA is based on using other molecular species such as hyaladherins or proteoglycan fragments that selectively recognize and bind HA [33]. The current HA assays are sensitive thus amenable to testing small biosamples and biopsies. Many sandwich‐like ELISA assays often faces challenges in detecting low MW HA (< 35 kDa) because they cannot effectively capture and detect smaller polymer chains that require a simultaneous binding to at least one HA binding protein (HABP) molecule on the surface, and one HABP detector molecule from the solution [34]. As HA degradation with disease or aging often occurs, the lower detection capability of the smaller MW species is especially problematic. Overall, a universal method for HA quantification that covers a wide range of sugar chain sizes in a sensitive fashion is highly desirable.

Analysis of disaccharides coupled with tandem mass (LC‐MS/MS) is one of the most widely used techniques for the total content determination of GAGs such as HA, heparan sulfate, and chondroitin sulfate [35, 36]. The LC‐MS/MS method has emerged as the preeminent standard approach for glycosaminoglycan analysis, offering superior sensitivity, specificity, and the capacity to generate unambiguous molecular signals, thereby addressing the inherent limitations of traditional LC‐UV techniques. Although the LC‐MS/MS method offers adequate precision, the absolute quantification of the ΔUAβ(1 → 3)GlcNAc disaccharide was difficult to achieve without an authentic internal reference standard [34]. The advancement in the synthesis of structurally homogeneous glycosaminoglycans using the chemoenzymatic approach has led to the improvement of the reparation of reference standards for the disaccharide analysis [37, 38]. Various ^13^C‐labeled authentic heparan sulfate and chondroitin sulfate disaccharides were used to improve an LC‐MS/MS‐based method [35, 36, 39]. Our innovative integration of a ¹³C‐labeled disaccharide calibrant ΔUAβ(1 → 3)GlcNAc not only enhances the method's analytical precision but also ensures unparalleled consistency in the quantification of hyaluronic acid across diverse and complex biological matrices [40, 41, 42].

## 2 Materials and Methods

### 2.1 Materials

Unlabeled HA ΔUAβ(1 → 3)GlcNAc disaccharide standard was purchased from Galen Molecular (North Haven, CT, USA). Sodium cyanoborohydride and 2‐aminoacridone (AMAC) were purchased from Sigma‐Aldrich (St. Louis, MO, USA). Q‐Sepharose resin was purchased from GE Healthcare (Chicago, IL, USA). HA ELISA Kit (Universal, 50–1600 ng/mL range) was purchased from Echelon Biosciences (Salt Lake City, UT, USA). Goat serum was purchased from Millipore Sigma (Burlington, MA, USA). All reagents and chemicals were high‐performance LC (HPLC) grade or LC‐MS grade.

### 2.2 Preparation of Recombinant PmHAS

Soluble active recombinant PmHAS^1–703^ enzyme was produced in *Escherichia coli* Xja host cells (Zymo Research Corp., Orange, CA) grown in Superior broth (Athena ES, Baltimore, MD) with ampicillin selection at 30°C. Synthase production was induced by adding isopropylthiogalactoside (0.2 mM final) at mid‐log phase. The phage lysin residing on the *E. coli* Xja chromosome was induced with l‐arabinose (3.3 mM final) present during the entire growth period. The temperature was reduced from 30°C to 22°C and the culture was maintained overnight. The cells were harvested by centrifugation and frozen at −80°C for future purification. Sonication was employed to release their cytoplasmic contents from the cells. The bacterial lysates were clarified by centrifugation at 15,000×*g* for 30 min. The purification of PmHAS^1–703^ was achieved using chromatography on Toyopearl Red AF resin (200 × 10 mm, Cytiva) (Tosoh Corp., Tokyo, Japan) using salt elution (50 mM HEPES, pH 7.2, 1 M ethylene glycol with 0–1.5 M NaCl linear gradient over 1 h) at a flow rate of 2 mL/min as described previously [43].

### 2.3 Preparation of UDP‐[^13^C]GlcA

The ^13^C‐labeled UDP‐GlcA was prepared from UDP‐[^13^C]Glc (UDP‐glucose) at gram‐scale by converting UDP‐Glc to UDP‐GlcA using UDP‐glucose dehydrogenase (UDGH) as described previously [44]. The preparation of UDP‐[^13^C]Glc was completed by incubating fully ^13^C‐labeled glucose (from Cambridge Isotope Laboratories) using two engineered *E. coli* strains expressing four enzymes, including glucokinase (GLK), phosphoglucomutase (PGM), UDP‐glucose pyrophosphorylase (UDPGP), and inorganic pyrophosphatase (PPA) [44]. [^13^C]Glucose and UTP (from Carbosynth) were converted to the product using a permeabilized bacterial culture system to prepare UDP‐[^13^C]Glc. UDP‐[^13^C]Glc was converted to UDP‐[^13^C]GlcA by UDGH, and the UDP‐[^13^C]GlcA product was purified using Q‐Sepharose. To determine the final concentration, the peak area at 260 nm of the [^13^C]‐labeled product was compared with known amounts of unlabeled UDP‐GlcA (Sigma) using an anion exchange HPLC.

### 2.4 Chemoenzymatic Synthesis of ^13^C‐Labeled HA Disaccharide Calibrant

The synthesis of ^13^C‐labeled ΔUAβ(1 → 3)GlcNAc disaccharide calibrant started from commercially available 4‐Nitrophenyl‐β‐d‐glucuronide (GlcA‐pNP). Seven elongation steps were performed to obtain GlcNAc‐[GlcA*‐GlcNAc]_3_‐GlcA‐pNP with hyaluronic acid synthase (PmHAS) from *Pasteurella multocida* where “GlcA*” indicates a ^13^C‐labeled GlcA residue. The elongation from GlcA‐pNP to GlcNAc‐GlcA‐pNP was carried out by incubating 5 mg GlcA‐pNP, 0.03 mM UDP‐GlcNAc, PmHAS (400 μg/mL) in a buffer solution containing 50 mM TRIS (pH 7.5) and 3 mM MnCl_2_ at 30°C overnight. In the following elongation step, the disaccharide substrate (GlcNAc‐GlcA‐pNP) was converted to GlcA*‐GlcNAc‐GlcA‐pNP by incubating with 0.03 mM UDP‐[^13^C]GlcA, PmHAS (400 μg/mL), 50 mM Tris (pH 7.5), and 3 mM MnCl_2_ in a total volume of 10 mL at 30°C overnight. The octamer backbone was completed through alternating addition of GlcNAc and GlcA* residues. The reaction degree was monitored by strong anion‐exchange chromatography on a Pro Pac PA1 column (particle size 10 μm, 9 mm i.d. × 250 mm, Thermo Fisher Scientific) with a 10%–25% gradient over 40 min (mobile phase A 20 mM NaOAc, pH 5.0; phase B 20 mM NaOAc, 1 M NaCl, pH 5.0) monitoring absorbance at 310 and 260 nm. The purification was performed using a C18 column (particle size 50 μm, 10 mm i.d. × 200 mm; Biotage). Electrospray ionization mass spectrometry (ESI‐MS) spectra and HPLC chromatograms are reported in Supporting Information S1: Figure S1A,B, respectively. Purified HA 8‐mer was subjected to complete digestion with chondroitin ABCase to isolate ^13^C‐labeled disaccharide. Enzymatic digestion was carried out with 10 mg of 8‐mer in 100 mM sodium acetate/2 mM calcium acetate buffer (pH 7.0) using 400 μL of 5 mg/mL chondroitin ABCase solution. The mixture was incubated at 37°C overnight to reach complete digestion. The ^13^C‐labeled disaccharide was then purified using a Pro Pac PA1 column (particle size 10 μm, 9 mm i.d. × 250 mm, Thermo Fisher Scientific). The column was equilibrated for 10 min 100% mobile phase A (20 mM NaOAc, pH 5.0) before injection, followed by isocratic elution at 100% mobile phase A for 5 min and followed by a linear gradient 0% to 25% mobile phase B (mobile phase B 20 mM NaOAc, 1 M NaCl, pH 5.0) over 40 min with monitoring absorbance at 232 nm. The purified disaccharide was then desalted by a GE Healthcare Tricorn column (10 mm i.d. × 200 mm) packed with Bio‐Gel P‐10 Media (particle size 45–90 μm) at a flow rate of 0.5 mL/min using pure water as mobile phase. After purification, the disaccharide was desalted on a Sephadex G‐10 column in water. The isolated ^13^C‐labeled ΔUAβ(1 → 3)GlcNAc disaccharide calibrant was then dried, weighted, and stored at −20°C. On a lab scale, the synthesis of disaccharide calibrants can be readily accomplished within a span of 10 days for quantities ranging from 1.5 to 50 mg. Moreover, just 1 mg of a disaccharide calibrant is sufficient to conduct approximately 10,000 analyses.

### 2.5 Preparing HA ^13^C‐Labeled Polysaccharides With Quasi‐Monodispersity

**Figure 1:**
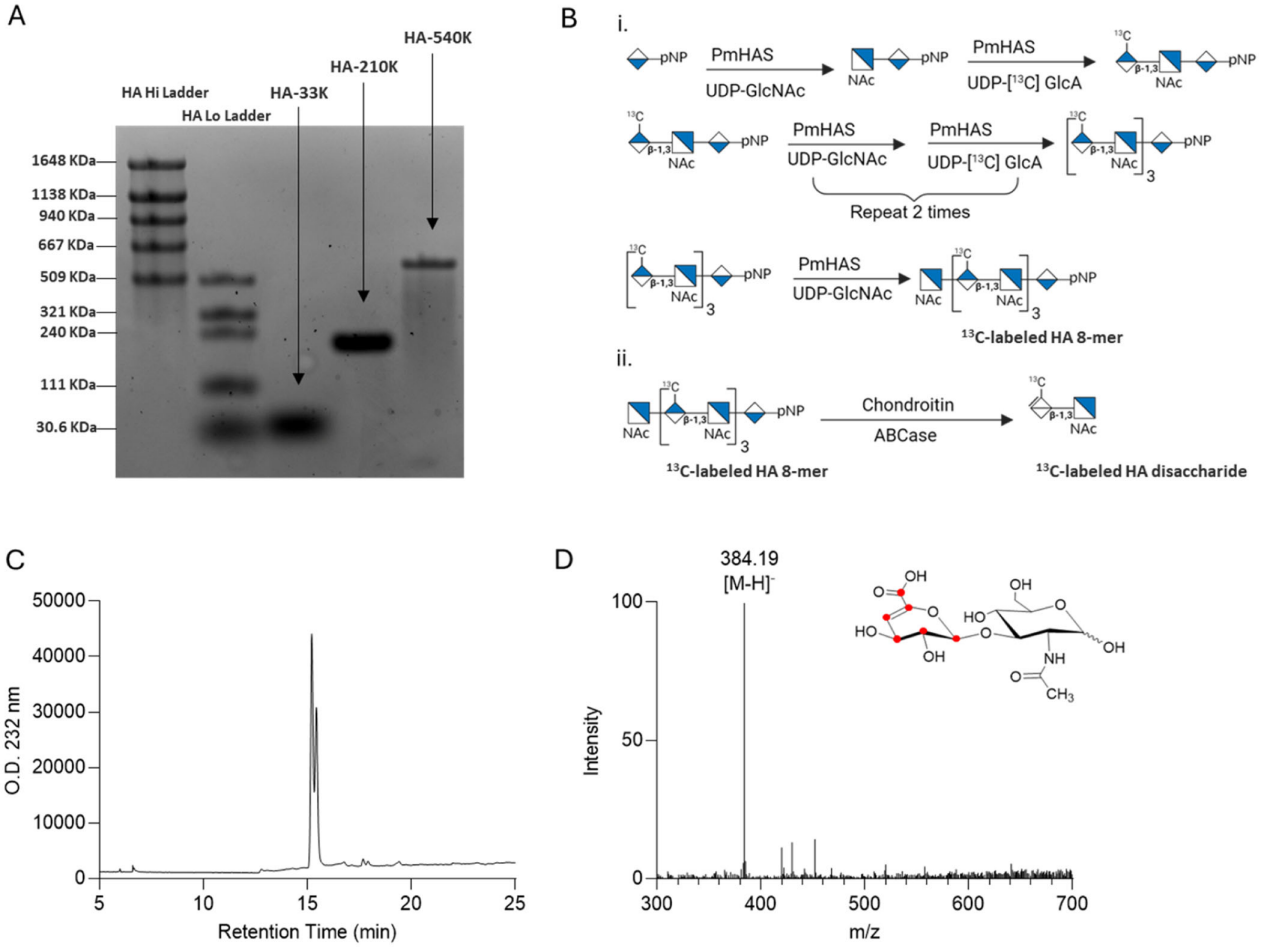
Characterization of synthetic HA polysaccharides and HA ΔUAβ(1 → 3)GlcNAc disaccharide. (A) Image of agarose gel (0.7%) analysis of three ^13^C‐labeled HA quasi‐monodisperse polysaccharides, HA‐35K, HA‐200K, and HA‐450K; about 4 mg of each sample was prepared. MW standards, Hyalose Select‐HA Low and High Ladders. (B) Top: scheme for the chemoenzymatic synthesis of ^13^C‐labeled HA 8‐mer. Bottom: scheme of the digestion of the HA 8‐mer with Chondroitin ABCase to obtain ^13^C‐labeled HA disaccharide calibrant. (C) Strong anion‐exchange chromatogram of HA disaccharide (retention time 15.4 min) with UV detector set at 232 nm. (D) ESI‐MS spectrum of ^13^C‐labeled HA disaccharide. Chemical structure is shown on top of figure; ^13^C atoms are denoted in red. The measured molecular weight (385.19 Da) is very close to the calculated value of (385.32 Da).

Three HA polysaccharides (HA‐33K, HA‐210K, and HA‐540K) were prepared using the synchronized stoichiometrically‐controlled enzymatic synthesis method [45, 46, 47, 48] employing the UDP‐^13^C‐GlcA and UDP‐GlcNAc donors. An unlabeled HA tetramer (two repeat units; derived from testicular hyaluronidase digestion [46]) acceptor was used to synchronize the extension process to yield narrow size distribution HA polymers. The stoichiometric ratio of UDP‐sugars to HA 4‐mer acceptor was employed to control the HA polymer target size. The HA products were purified by using sequential heat treatment (95°C, 1 min, clarification by centrifugation, and ultrafiltration with 3 kDa cutoff spin columns (Millipore) to both remove unreacted materials and desalt. The MW distribution of the HA polysaccharides was assessed with a 1X TAE 0.7% agarose gel and Stains‐All detection [49] and HA standards with MWs determined by multi‐angle light scattering as shown in Figure 1A. The estimated MW for the three HA polysaccharides was 33 kDa (~82 disaccharide repeats), 210 kDa (~525 disaccharide repeats), and 540 kDa (~1350 disaccharide repeats) for HA‐33K, HA‐210K, and HA‐540K, respectively. The concentration of HA was measured based on the carbazole assay with a GlcA standard [50].

### 2.6 Tissues Harvested From Healthy or Acetaminophen (APAP)‐Injured Mice

Tissues, including brain, heart, kidneys, lungs, liver, and plasma were dissected from five healthy adult male mice (10 weeks old). Additional livers (*n* = 5) were obtained from APAP‐induced injured liver mice. To induce liver failure, fresh APAP was dissolved in warm (~50°C) sterile 0.9% sodium chloride solution (sterile saline), cooled to 37°C, and injected intraperitoneally at 400 mg/kg. The extent of the liver damage was assessed by measuring the plasma level of alanine transaminase (ALT) [51]. All animal experiments were approved by the Institutional Animal Care and Use Committee (IACUC) of the University of North Carolina at Chapel Hill (Chapel Hill, NC).

### 2.7 Labeling Disaccharide With AMAC

Before tandem mass analysis, AMAC derivatization was carried out. The ΔUAβ(1 → 3)GlcNAc disaccharide was labeled using 5 μL of 0.1 M AMAC in DMSO/glacial acetic acid solution (17:3, v/v) and incubated at room temperature for 15 min. Then 5 μL of 1 M aqueous sodium cyanoborohydride (freshly prepared) was added. The mixture was incubated at 45°C for an additional 2 h and then clarified by centrifugation (21,300×*g*, 3 min) to obtain the supernatant for the LC‐MS/MS analysis.

### 2.8 LC–MS/MS Analysis

The analysis of AMAC‐labeled ΔUAβ(1 → 3)GlcNAc disaccharide was performed on a Vanquish Flex UHPLC System (Thermo Fisher Scientific) coupled with TSQ Fortis triple‐quadrupole mass spectrometry as the detector using an Agilent Poroshell 120 EC‐18 reverse phase column (2.7 μm, 4.6 × 50 mm) at 45°C at 0.3 mL/min. Mobile phase A was 50 mM ammonium acetate aqueous solution and mobile phase B was methanol. The column was equilibrated with 5% buffer B for 20 column volumes. The elution gradient was buffer B from 5% to 45% for 10 min, ramped to 100% in 0.2 min, then target analytes were eluted with 100% buffer B for 4 min. Online triple‐quadrupole mass spectrometry operating in the multiple reaction monitoring (MRM) mode was used as the detector. The MRM transition of each component is shown in Supporting Information S1: Table S1. Ionization was set in the negative mode and the ion source parameters are as follows: spray voltage 4,000 V, sheath gas 45 Arb, Aux gas 15 Arb, sweep gas 0 Arb, vaporizer temperature 350°C, and ion transfer temperature 320°C. TraceFinder software (v. 4.1; Thermo Scientific) was used for data processing.

### 2.9 NMR Analysis of HA Disaccharide

HA ΔUAβ(1 → 3)GlcNAc disaccharide (0.5 mg) was dissolved in 0.6 mL D_2_O (99.996%, Sigma‐Aldrich) and lyophilized three times to remove the exchangeable protons. The samples were re‐dissolved in 0.6 mL D_2_O and transferred to NMR microtubes (O.D. 5 mm, Norrell). The first NMR experiment was performed on Bruker Advance 850 MHz spectrometer equipped with cryoprobe. The temperature was set at 30°C (303 K) to avoid signal overlapping between H_2_O with the β‐anomer of GlcNAc. 1D ^1^H‐NMR and 2D ^1^H‐^13^C HSQC were carried out under the conditions described previously [52, 53] to characterize HA disaccharide (Supporting Information S1: Figure S2) for further analysis involving controlled degradation. NMR tube was kept at 30°C for 48 h and then analyzed again using the same conditions to determine the rate of degradation of the ΔUAβ(1 → 3)GlcNAc starting material and the formation of “peeling by‐products.”

### 2.10 HA Extraction From Mice Tissues and Quantitation Analysis

Five different tissues, including brain, heart, kidneys, liver, and lungs, were collected from healthy adult mice for quantification of the total HA content. The HA extraction from mice tissues was conducted with excision, homogenization, and defatting by suspension and vortexing in a series of chloroform/methanol mixtures (2:1, 1:1, and 1:2 (v/v)). The defatted tissues were dried and weighed to obtain the dry weight and subjected to Pronase E digestion (10 mg:1 g (w/w), Pronase E/tissue) at 55°C for 24 h to degrade the proteins. Hyaluronic acid was recovered from digested solution using Q‐Sepharose column. Although the current method successfully separates larger HA oligosaccharides, it is not capable of detecting 4‐mer or 6‐mer, as these shorter fragments do not bind to the Q‐Sepharose column and are thus excluded from the analysis. Consequently, a new approach is required to accurately measure these very short HA oligosaccharides, which is beyond the scope of this study. Q‐Sepharose mobile phase A was 20 mM Tris, pH 7.5 and 50 mM NaCl, and mobile phase B was 20 mM Tris, pH 7.5, and 1 M NaCl. After loading the digested solution, the column was washed with 1.5 mL mobile phase A, followed by 1.5 mL mobile phase B to elute the HS fraction. A 3‐kDa centrifugal filter (Millipore) was used to desalt the hyaluronic acid eluting from the Q‐Sepharose column by performing three rounds of ultrafiltration with deionized water to remove salt. After freeze‐drying, the desalted material was resuspended in 50 mM MOPS (pH 7.0) and incubated with 5 μL of 5 mg/mL Chondroitin ABCase solution at 37°C for 4 h. The ΔUAβ(1 → 3)GlcNAc disaccharide was recovered by centrifugation, using a 3‐kDa centrifugal filter to remove the enzyme and other non‐digested GAGs (heparan sulfate). A known amount of ^13^C‐labeled disaccharide (100 ng/sample) was added as a mass calibrant to the digestion solution and the mixture freeze‐dried before AMAC derivatization. The procedures for AMAC labeling and LC‐MS/MS analysis of the collected disaccharides were performed as described above.

### 2.11 Isolation and Quantification of HA From Plasma

HA was extracted the plasma samples with methanol‐induced protein precipitation, proteinase digestion, Q‐Sepharose column purification, and Chondroitin ABCase depolymerization. Plasma samples (200 μL) were mixed with 1 mL of methanol, vortexed for 1 min, and proteins were precipitated at room temperature for 10 min. The sample was centrifuged at 18,000×*g* for 10 min and the supernatant was discarded. The pellet was then digested with Pronase E (10 mg:1 g (w/w), Pronase E/protein) at 55°C for 24 h. HA was isolated from the mixture using a micro Q‐Sepharose column with the procedure mentioned above. The eluted HA was then desalted and consequently digested overnight using chondroitin ABCase. The digestion solution was filtered to collect HA ΔUAβ(1 → 3)GlcNAc disaccharide to perform quantification after the ^13^C‐labeled disaccharide calibrant was added and AMAC conjugation carried out.

### 2.12 HA ELISA‐Like Assay

The manufacturer's protocol (K‐1200; Echelon Biosciences, Inc.) was followed. Briefly, the assay was initiated by incubating the mixture of the HA detector and samples, standards, or controls in a plate pre‐coated with HA for competitive binding at 4°C. After 1 h, an alkaline phosphatase‐linked antibody was added to the plate and then incubated at 37°C for 30 min, followed by a pNPP (p‐Nitrophenyl phosphate) colorimetric reaction in the dark for 30 min at room temperature after which the stop solution was added to terminate the assay. Between each step above, unbound reagents in the wells were removed by a series of washing steps. The sample's HA concentration was calculated by comparing their absorbance at 405 nm with the HA standard curve under a sigmoidal dose response‐variable slope model. To compare the detectable range between LC‐MS and ELISA, each HA polysaccharide stock solution (1.5, 1.3, and 1.5 mg/mL, respectively) was diluted in the sample dilution buffer to produce a dilution series of 10,000, 1000, 100, and 10 ng/mL (note: the 10,000 and 10 ng/mL points were out of the ELISA kit's range). Every sample was measured in three replicate wells and the results were averaged for data presentation.

## 3 Results and Discussion

### 3.1 Enzymatic Synthesis of ^13^C‐Labeled HA Disaccharide and ^13^C‐Labeled HA Polysaccharide

We prepared various HA species with the GlcA residues being completely ^13^C‐labeled: three quasi‐monodisperse HA polysaccharides, an HA octamer, and an HA ΔUAβ(1 → 3)GlcNAc disaccharide derivative for this study. For the polysaccharides, the acceptor:donor ratio in the polymerization was used to control the target HA polysaccharide size [46]. Molecular weight estimates were obtained by comparing the relative migration on an agarose gel to HA standards of known masses (Figure 1A). A defined HA octamer (four repeats) was produced by a stepwise extension process (Figure 1B).

The reaction scheme for the preparation of ^13^C‐labeled HA disaccharide from the HA octamer is shown in Figure 1B. The 8‐mer was then subjected to the digestion of chondroitin ABCase to yield ^13^C‐labeled HA disaccharide. The ΔUAβ(1 → 3)GlcNAc disaccharide showed a doublet UV peak from the HPLC analysis due to the fact it is a mixture of two anomeric forms. The MW of the HA disaccharide was measured to be 385.19 Da, very similar to the calculated MW of 385.32 Da. Here, a purified synthetic HA 8‐mer made by step‐wise synthesis was employed to generate the first calibrant; in principle, however, potentially any chemoenzymatically synthesized HA polymer made with the ^13^C‐labeled sugar donor can be isolated and digested with lyase to yield the identical ^13^C‐disaccharide calibrant.

### 3.2 Chemical Stability of ^13^C‐Labeled HA Disaccharide

**Figure 2:**
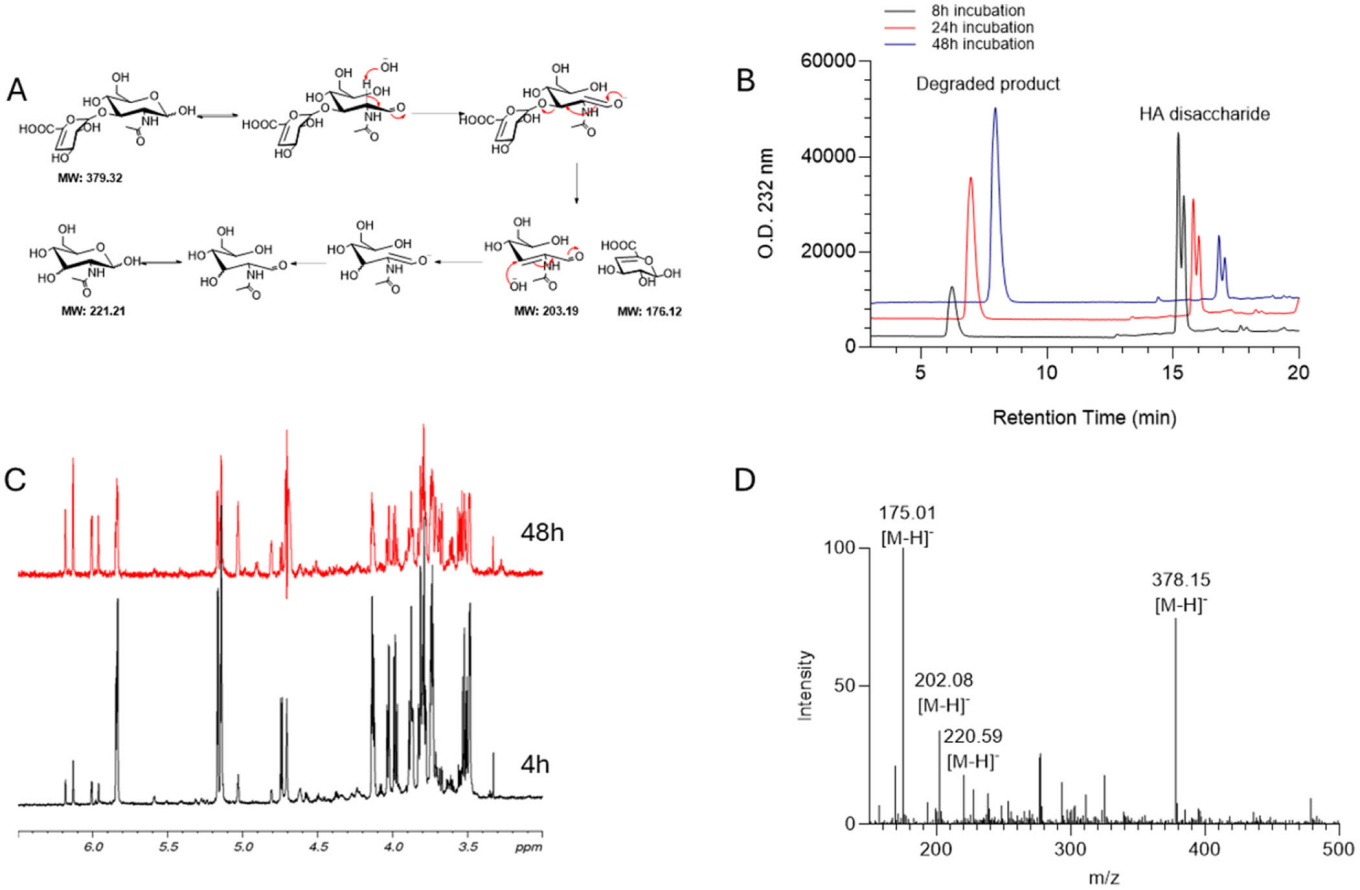
Stability of ^13^C‐labeled HA ΔUAβ(1 → 3)GlcNAc disaccharide. (A) Proposed peeling reaction mechanism of 1,3‐linked HA disaccharide. Under basic conditions, the GlcNAc monosaccharide at the reducing end will undergo through β‐elimination to release the substituent in 3‐position. (B) HPLC chromatogram of HA disaccharide incubated at 37°C in 50 mM Na_2_HPO_4_, pH 9.0 for different time points. (C) 1H‐NMR spectra of HA disaccharide in D_2_O at different time points at 30°C (303 K): 4 h (black, bottom) or 48 h (red, top). Anomeric signals at 5.2 ppm belonging to the disaccharide structure decreased during time in favor of signals at higher frequencies (6–6.2 ppm) presumably related to the unsaturated GlcA monosaccharide. (D) ESI‐MS analysis of partially degraded material was performed to characterize all the species. HA ΔUAβ(1 → 3)GlcNAc disaccharide and both glucuronic acid and N‐acetylglucosamine were found.

As a reference standard, it is important to demonstrate that the ΔUAβ(1 → 3)GlcNAc calibrant is stable under the experimental conditions. We investigated the stability of ^13^C‐labeled disaccharide under different conditions. Although GAGs disaccharides are generally stable, heparan sulfate oligosaccharides with a 3‐*O*‐sulfated glucosamine residue at the reducing ends have been previously reported for its susceptible to degradation under basic conditions [54, 55]. Peeling reaction is a base‐catalyzed β‐elimination that also occurs at the reducing end of 1,3‐linked glycans, a structural feature in HA disaccharide. We examined the stability of the ΔUAβ(1 → 3)GlcNAc disaccharide standard under different conditions focusing our attention on the impact from different temperatures and pH values (Table 1). The tested environments included acidic (50 mM NaH_2_PO_4_, pH 4.7), neutral (50 mM MOPS, pH 7.0), and basic (50 mM Na_2_HPO_4_, pH 9.0) conditions. These “stressed” samples were subjected to the high‐resolution anion‐exchange HPLC analysis after different incubation periods. A slight degradation at 25°C was observed at the basic condition after 24 h. No obvious degradation was observed at acidic or neutral pH (Table 1). The stability test conducted at 37°C, however, showed an increase in degradation exclusively at pH 9.0 (Figure 2B and Table 1). We discovered that using MOPS at neutral pH is the best condition to minimize the degradation of HA disaccharide. We further investigated the degradation of HA disaccharide at a higher temperature mimicking the chondroitin ABCase digestion. To this end, HA disaccharide was dissolved in the digestion buffer containing 50 mM NaAcO, pH 6.0, 1 mM Ca(AcO)_2_, 0.1 g/L bovine serum albumin. The mixture was heated at 95°C for 10–60 min. High‐resolution anion‐exchange HPLC showed an extensive degradation after 10 min and a complete degradation after 60 min (Supporting Information S1: Figure S3). ^1^H‐NMR and MS analysis were performed (respectively, Figure 2C,D) to confirm that the rationale behind our proposed mechanism of peeling degradation (Figure 2A) was correct. The intensity of some specific NMR signals of the HA disaccharide substantially decreased after 48 h at 30°C. Due to the extensive signal overlap in the 3.4–4.2 ppm region, we monitored a few isolated signals belonging to the anomeric protons of GlcNAc (α and β anomers at 5.14 and 4.74 ppm, respectively), GlcA (5.16 ppm), and H4 of GlcA (5.82 ppm). A clear sign of degradation was the lower intensity of these peaks while increasing intensity of new peaks located at higher frequencies (between 6 and 6.2 ppm), presumably belonging to the unsaturated GlcA monosaccharide after peeling reaction. Moreover, the region between 3.4 and 4.2 ppm appeared even more crowded with several new signals appearing especially between 3.5 and 3.7 ppm. Three molecular ions were detected at the *m*/*z* values of 175.01, 202.08, and 220.59 Da from the LC‐MS analysis, representing the degraded fragments from the HA ΔUAβ(1 → 3)GlcNAc disaccharide. Based on our results, the sample heating step at 95°C for 10 min to terminate the digestion of HA with chondroitin ABCase would cause the degradation of the ΔUAβ(1 → 3)GlcNAc disaccharide, thereby, the heating step was avoided from our subsequent analysis. In the revised protocol for the analysis of HA from biological sources, we decided to use MOPS buffer at pH 7.0 as incubation buffer and avoid the boiling step to stop digestion. This treatment improved the sample recovery for the subsequent disaccharide analysis and offered more consistent results.

**Table 1:** Percentage of HA ΔUAβ(1 → 3)GlcNAc disaccharide (2‐mer) degradation under different pH and temperature conditions. HA disaccharide showed chemical instability in basic condition and at higher temperature.

		NaH_2_PO_4_ pH 4.7				MOPS pH 7				Na_2_HPO_4_ pH 9			
Temperature	Compound	0 h	8 h	24 h	48 h	0 h	8 h	24 h	48 h	0 h	8 h	24 h	48 h
25°C	HA 2‐mer	100%	99%	98%	98%	100%	98%	98%	98%	100%	79%	69%	60%
	Peeling by‐product	0%	1%	2%	2%	0%	2%	2%	2%	0%	21%	31%	40%
37°C	HA 2‐mer	100%	99%	98%	97%	100%	99%	97%	94%	100%	43%	28%	18%
	Peeling by‐product	0%	1%	2%	3%	0%	1%	3%	6%	0%	57%	72%	82%

### 3.3 Validation of the LC‐MS/MS Method for the Analysis of HA Polysaccharides

A series of validation experiments were carried out to demonstrate reliability of our LC‐MS/MS method. Three HA polysaccharides with quasi‐monodisperse molar masses were used to compare the ability of detection and quantification of ELISA‐like assay and LC‐MS/MS with internal ^13^C‐ΔUAβ(1 → 3)GlcNAc calibrant. The HA‐33K, HA‐210K, and HA‐540K were synthesized by the enzymatic method as described above. The concentration of the three size‐defined HA polysaccharides was determined by carbazole assay using GlcA as a reference (Table 2). LC‐MS/MS was performed to measure the concentration of three HA polysaccharides with values ranging from 1.31 to 1.51 mg/mL (Supporting Information S1: Table S2), very close to the HA concentration obtained by the carbazole assay. Data from ELISA‐assay quantification (Standard curve in Supporting Information S1: Figure S4), except for HA‐210K that showed a ~2‐fold decrease in terms of concentration, are comparable with both carbazole assay and LC‐MS (Table 2).

**Table 2:** Comparison of HA polysaccharides concentration calculated with ELISA assay and LC‐MS/MS.

Compound	Anticipated (mg/mL)^a^	LC‐MS/MS (mg/mL)^b^	ELISA (mg/mL)^b^
HA‐33K	1.5	1.47 ± 0.06	1.5 ± 0.06
HA‐210K	1.3	1.38 ± 0.04	0.75 ± 0.06
HA‐540K	1.5	1.41 ± 0.05	1.2 ± 0.22

^a^ Based on the carbazole assay for GlcA.

^b^ Data are presented as an average of three determinations ± SD.

**Figure 3:**
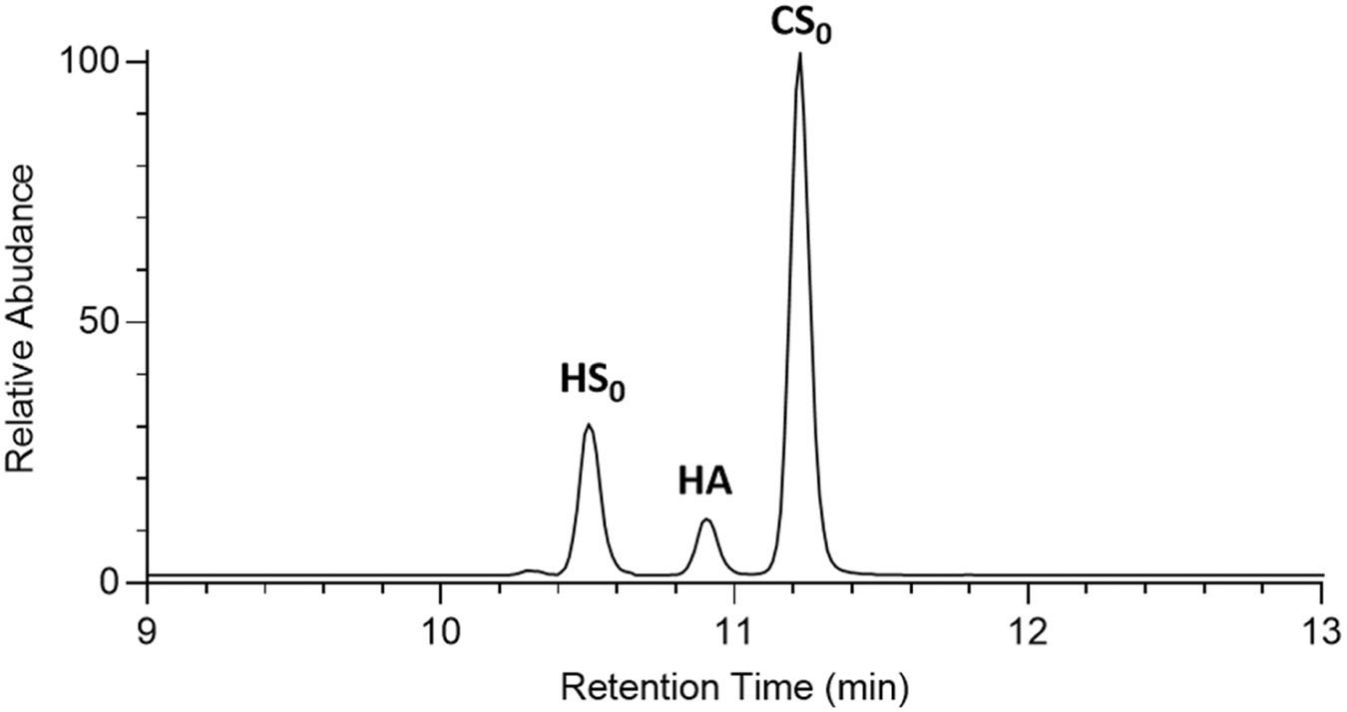
EIC (Extract Ion Chromatogram) of AMAC‐labeled disaccharides. The AMAC‐labeled unsulfated disaccharides standards from HS_0_ (ΔUAβ(1 → 4)GlcNAc), HA (ΔUAβ(1 → 3)GlcNAc), and CS_0_ (ΔUAβ(1 → 3)GalNAc) were resolved by LC‐MS analysis.

During the disaccharide analysis of biological samples, three different glycosaminoglycans yield distinct non‐sulfated disaccharides: ΔUAβ(1 → 3)GlcNAc from HA, ΔUAβ(1 → 4)GlcNAc from HS, or ΔUAβ(1 → 3)GalNAc from CS. All three disaccharides display an identical molecular weight thus indistinguishable by simple mass spectroscopy without fragmentation studies. To avoid potential misidentification among these disaccharides during the analysis, we must demonstrate our LC method can resolve this trio of lyase‐derived fragments because many tissues can contain all three GAG species simultaneously. As predicted, we successfully separated the three different disaccharides by LC as shown in Figure 3. Notably, by not using heparin lyases, we ensured that the presence of HS does not interfere with the quantification of HA, and even in the presence of disproportionately higher amounts of CS, our method reliably distinguished the signals, confirming its robustness under typical biological conditions.

**Figure 4:**
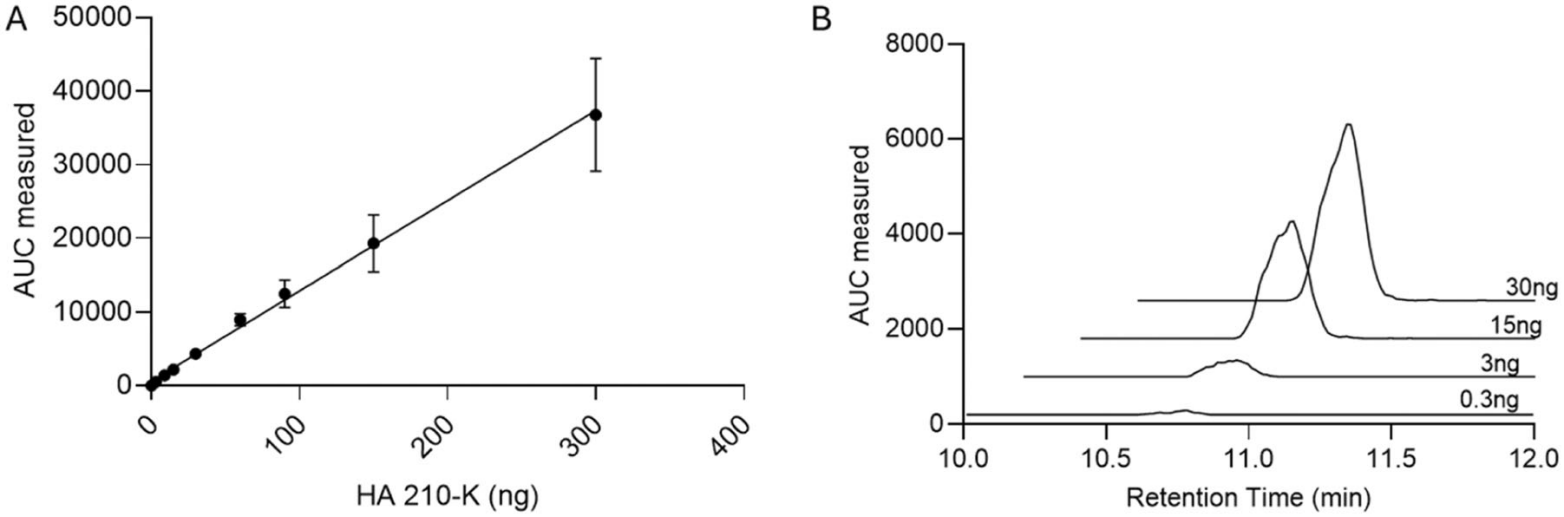
LC‐MS/MS quantification. (A) LOD and linear response of LC‐MS/MS method using HA 210‐K (*R* ^2^ = 0.955). Data are presented as mean (*n* = 3). (B) Comparison of AUCs (area under the curve) obtained with different HA 210‐K amounts.

**Figure 5:**
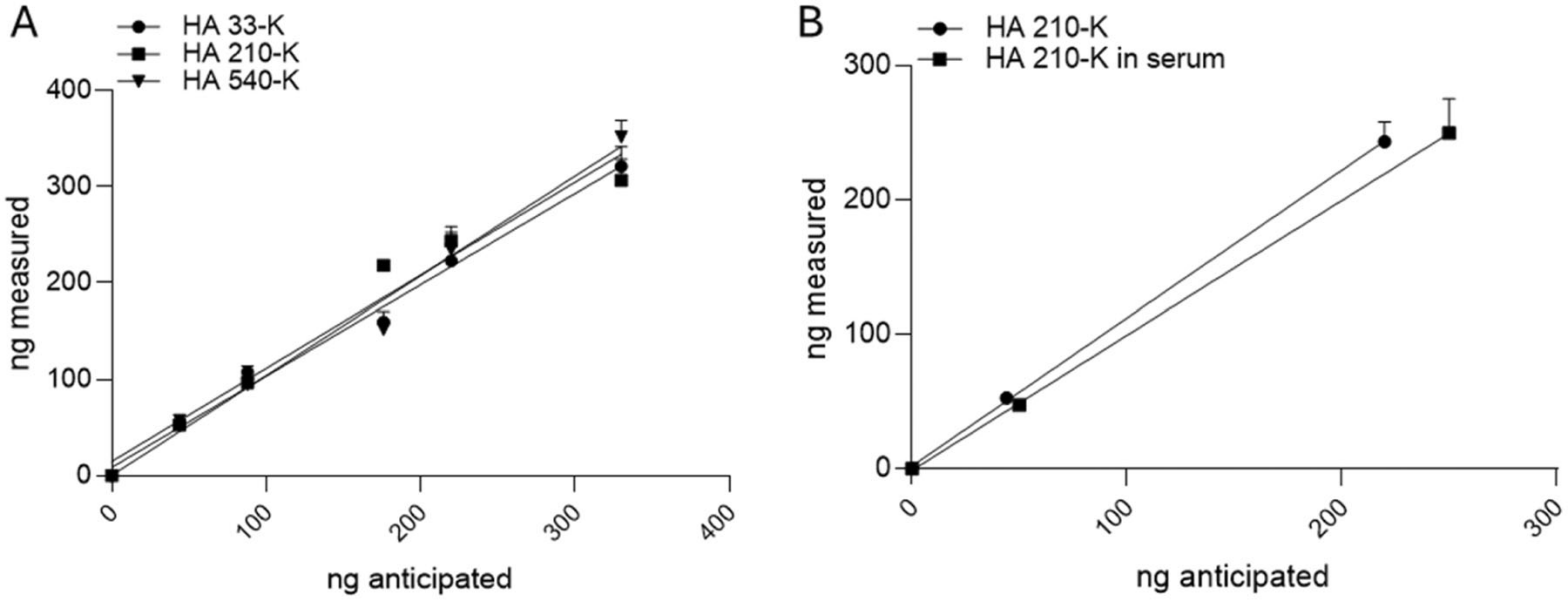
Linearity of LC‐MS/MS quantification. (A) Linear response of the ΔUAβ(1 → 3)GlcNAc derived from HA 33‐K (*R* ^2^ = 0.984), HA 210 K (*R* ^2^ = 0.961), and HA 540 K (*R* ^2^ = 0.979) polysaccharides quantified by LC‐MS/MS. (B) Comparison of detection response of the LC‐MS/MS method of 210‐kDa HA when the polymer is dissolved in water (circles) (*R* ^2^ = 0.995) or goat serum (squares) (*R* ^2^ = 0.991). All data are presented as the mean (*n* = 3), and error bars indicate SD.

We further determined the ranges of the linear response from the LC‐MS/MS analysis (Figure 4A,B). Here, we present the data for HA‐210K as a representative. The LC‐MS/MS method displayed excellent linearity from 3 to 300 ng, covering 100‐fold of the expected analyte concentration. The estimated limit of detection for HA disaccharide was 200 pg with a signal‐to‐noise ratio of 3:1. The next validation experiment was to compare the amount of measured different HA polysaccharides with the anticipated amount in water as well as in serum to represent a more “physiological” biospecimen. For the different sizes of HA tested in our experiment, the measured amount correlated well with the anticipated amount (Figure 5A). Furthermore, we compared the amount of HA‐210K in water or in goat serum which roughly mimics the conditions to measure HA from mammalian plasma or its derivatives. As shown in Figure 5B, the measurement of the HA content both in water and in serum was very close at three input concentrations, representing potential low, medium, and high concentrations of HA in the blood. The method employs rigorous sample purification and an excess of chondroitin ABCase (2.5 U) to ensure complete degradation of hyaluronic acid, even in the presence of potential inhibitors. This approach is validated by successful measurements of HA in mouse tissues and plasma, confirming the proper function of chondroitin ABCase in the analysis.

### 3.4 Analysis of HA From Healthy Mice and APAP‐Induced Liver Injury Mice

**Figure 6:**
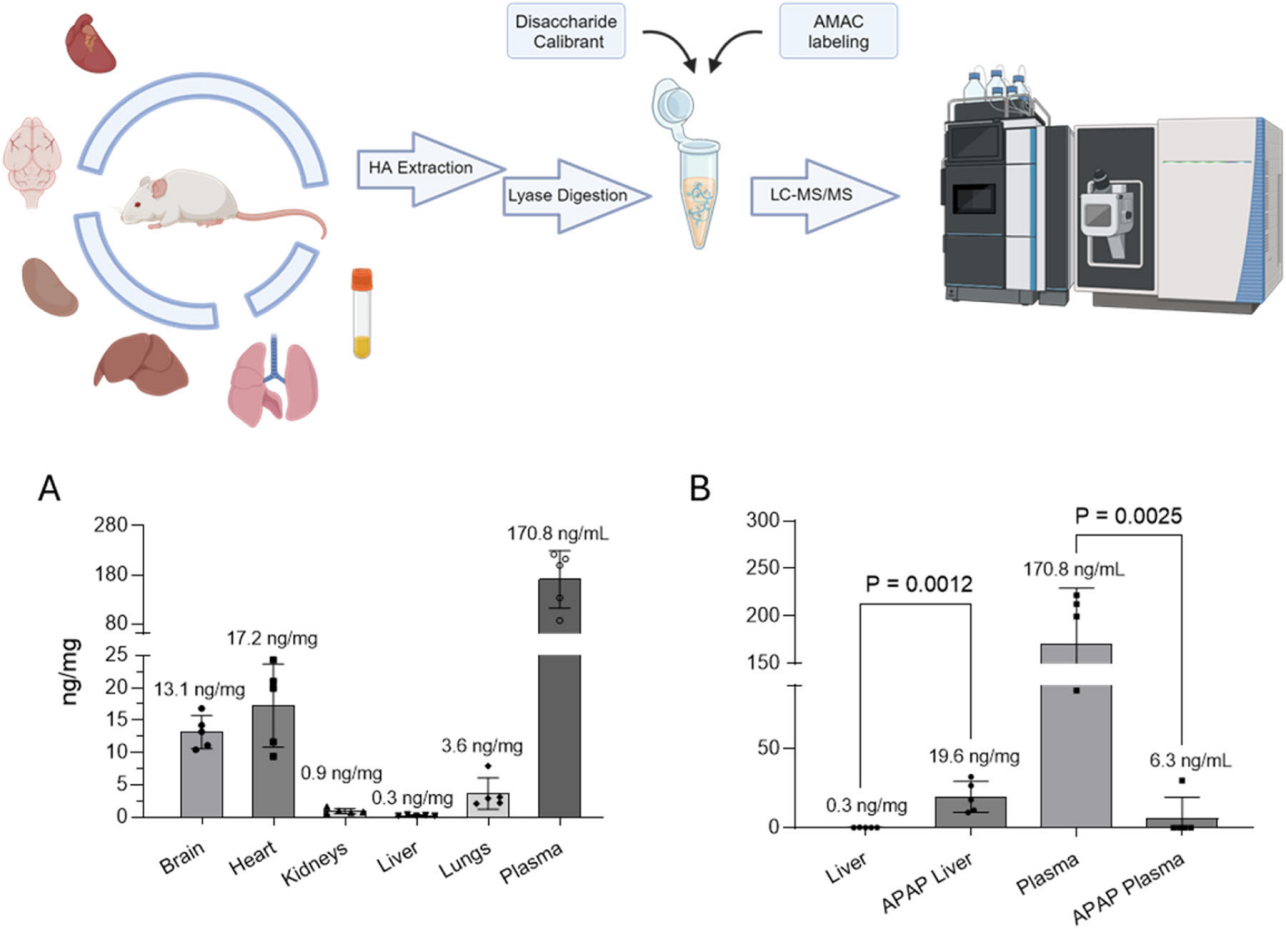
HA quantification from mice tissues. (Top) Workflow chart for the analysis. (A) Total content of HA from different mice tissues and fluids measured by LC‐MS/MS method using ^13^C‐labeled HA disaccharide calibrant. Total amount of HA is measured in ng/mg of dry tissue. For plasma samples the total amount is expressed in ng/mL. Single values, mean, and SD are shown in Supporting Information S1: Table S3. (B) Comparison of HA total content in liver and plasma from healthy and APAP‐injured mice. After liver failure induction, HA content is significantly lower in plasma, but a 75‐fold increase in liver was observed. The *p* value was determined by two‐tailed unpaired *t* test, and the values are indicated.

We employed the method in analyzing the HA from different biological sources. Here we performed the analysis of various tissues and plasma from healthy adult mice. In addition, we also analyzed the level of HA from the livers of mice suffering with acute liver injury induced by acetaminophen overdose. To this end, defatted and protein‐free samples were subjected to chondroitin ABCase digestion. After enzymatic digestion HA ^13^C‐labeled ΔUAβ(1 → 3)GlcNAc disaccharide was added to the digestion mixture before the LC‐MS/MS analysis. Although our method enables the determination of the amount of HA, it is incapable of measuring the sizes of HA. Despite the limitation, our data indicate that the highest concentration of HA was found in the plasma at 171 ± 58 ng/mL (*n* = 5) while the lowest concentration of HA was found in kidneys with 0.3 ng/mg of dry tissue weight (Figure 6A). Notably, we discovered a ~75‐fold increase in the level of liver HA in the APAP‐injured mice coinciding with a significantly lower amount of HA from APAP‐injured mice plasma (Figure 6B). It is known that HA plays a key role in inflammation both initiating inflammatory response or promoting recovery from tissue injury [56, 57]. This large increase in liver HA content in the injured mice may offer a new clue to investigate the physiological response at the localized inflammation processes. Furthermore, this increased HA signal could be potentially used as a biomarker for liver disease. Two potential hypotheses for the increased HA levels in the liver in this disease are that (a) the HA metabolism in the drug‐injured animals is perturbed such that fragments from the plasma are internalized (a function of the healthy liver), but not cleaved efficiently (i.e., lysosomes normally degrade HA polymer to monosaccharides which are not detectable with this or other HA identification methods), and/or (b), the damaged liver directly makes excess HA polymer as part of the damage or inflammation process.

## 4 Conclusions

In this manuscript, we reported a practical method to detect HA in biological sources. The crucial innovation in our approach is to include a ^13^C‐labeled ΔUAβ(1 → 3)GlcNAc disaccharide calibrant, which adds the capability to quantify HA content in biological specimens. The method involves the use of standard LC‐MS equipment commonly found in many modern biochemical core facilities. The ^13^C‐labeled HA disaccharide calibrant can be synthesized in large scale, making this standard widely accessible. In comparison with the commercially available ELISA kit for HA, our method offers a direct measurement of the disaccharide unit of HA with high specificity. One interesting finding is from the use of our method to analyze HA from the liver and plasma of healthy and APAP‐injured mice. This large increase in the level of HA in APAP‐injured mice liver and the consequent large depletion from plasma has not been reported previously.

Our next step will be to employ this method for the analysis of HA in different disease animal models and human patient samples to search for a new biomarker for biological studies. A robust and universal technique offers a valuable tool for advancing our understanding of the complexities of the roles of HA in health and disease.

## Author Contributions

E.S. developed the analytical method, completed synthesis, and the NMR and MS products analysis, and wrote the manuscript. D.E.G. conducted the synthesis of the hyaluronic acid polysaccharide and product characterization. K.A. conducted the mice experiment and tissues collection. J.Z. performed the ELISA assays. D.K. provided the chemical degradation mechanism. P.L.D., J.L., E.S., and D.E.G. prepared the enzymes for completing the synthesis. J.L. designed the project and wrote the manuscript. All authors contributed to the manuscript writing and approved the manuscript. All authors have reviewed and approved the final manuscript and agree with its content.

## Conflicts of Interest

J.L. is a founder and the chief scientific officer for Glycan Therapeutics. K.A. is a founder of Glyco Discoveries, a subsidiary of Glycan Therapeutics. Both J.L. and K.A. have equity in Glycan Therapeutics. P.L.D. has commercialized the quasi‐monodisperse HA reagent production and receives financial compensation for Hyalose, LLC products licensed by Echelon Biosciences, Inc. The other authors declare no conflicts of interest.

## Supporting information

Supporting information.

## Data Availability

All data supporting the findings of this study are available within the article and its supporting information.
