# Supplementary material for: Utility of Authentic 13C‐Labeled Disaccharide to Calibrate Hyaluronan Content Measurements by LC‐MS
Source: Proteoglycan Res. 2024 Nov 5;2(4):e70010. doi: 10.1002/pgr2.70010 (PMC11582344; doi:10.1002/pgr2.70010)
Supplement: Supplementary file 1 — Supporting information. [file PGR2-2-e70010-s001.docx]

**Utility of Authentic ^13^C-Labeled Disaccharide to Calibrate Hyaluronan Content Measurements by LC-MS**

Supplementary Materials

Eduardo Stancanelli^1^, Dixy E. Green^2^, Katelyn Arnold^1^, Jianxiang Zhang^1^, Deyu Kong^1^, Paul L. DeAngelis^2^ and Jian Liu^1, *^

1. Division of Chemical Biology and Medicinal Chemistry, Eshelman School of Pharmacy, University of North Carolina, Chapel Hill, North Carolina, USA.
2. Department of Biochemistry & Physiology, The University of Oklahoma Health Science Center, Oklahoma City, Oklahoma, USA. 73126

Authors: [eduardos@ad.unc.edu](mailto:eduardos@ad.unc.edu) 0000-0002-6006-2209

[dixy-green@ouhsc.edu](mailto:dixy-green@ouhsc.edu) 0000-0002-6237-0889

[arnoldk2@email.unc.edu](mailto:arnoldk2@email.unc.edu) 0000-0002-1750-125

[jianxz@unc.edu](mailto:jianxz@unc.edu) 0000-0002-1963-456X

[deyu0103@gmail.com](mailto:deyu0103@gmail.com) 0000-0002-9154-5967

[paul-deangelis@ouhsc.edu](mailto:paul-deangelis@ouhsc.edu) 0000-0002-8843-1344

Corresponding author: [jian_liu@unc.edu](mailto:jian_liu@unc.edu) 0000-0001-8552-1400


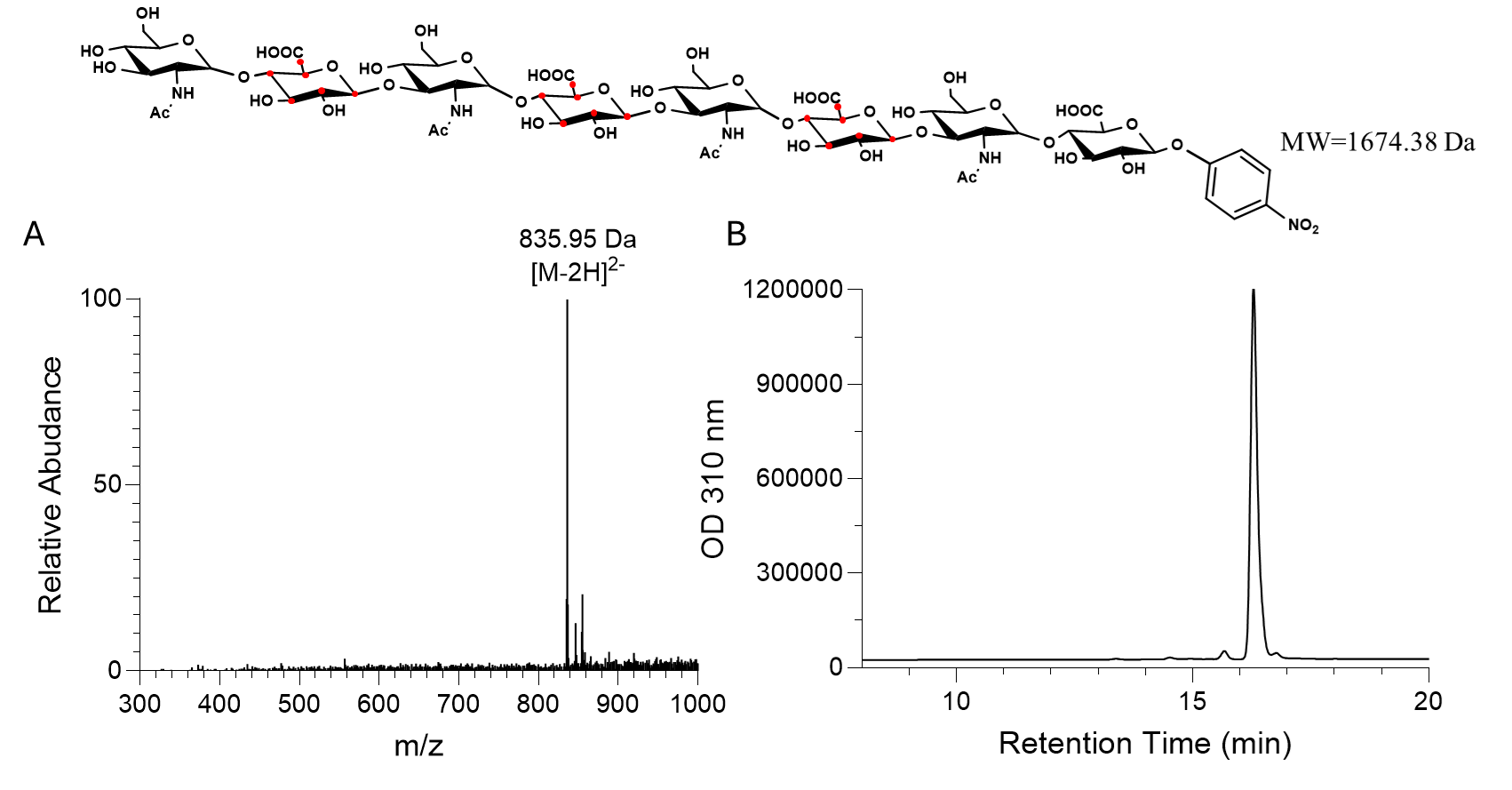


**Supplementary Figure S1**. HPLC and ESI-MS analysis of **HA 8-mer (4 repeats).** Panel A, the strong anion exchange-HPLC chromatogram of **HA 8-mer**. Panel B, the ESI-MS spectrum of **HA 8-mer.** Chemical structure is reported on top of the figure; the red spheres represent the ^13^C atoms.


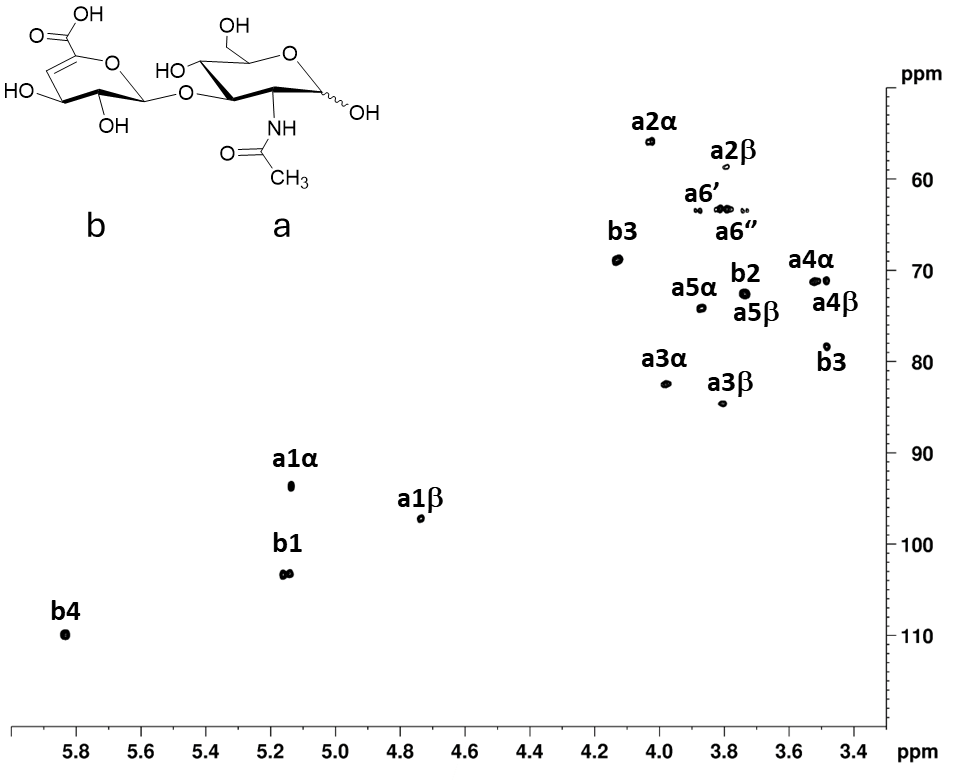


**Supplementary Figure S2.** ^1^H -^13^C HSQC spectrum of lyase-derived HA disaccharide (850 MHz, D_2_O). Chemical structure of HA disaccharide is shown on the top of figure denoting the ‘a’ and ‘b’ sugars; signals are indicated for each of the ring positions.


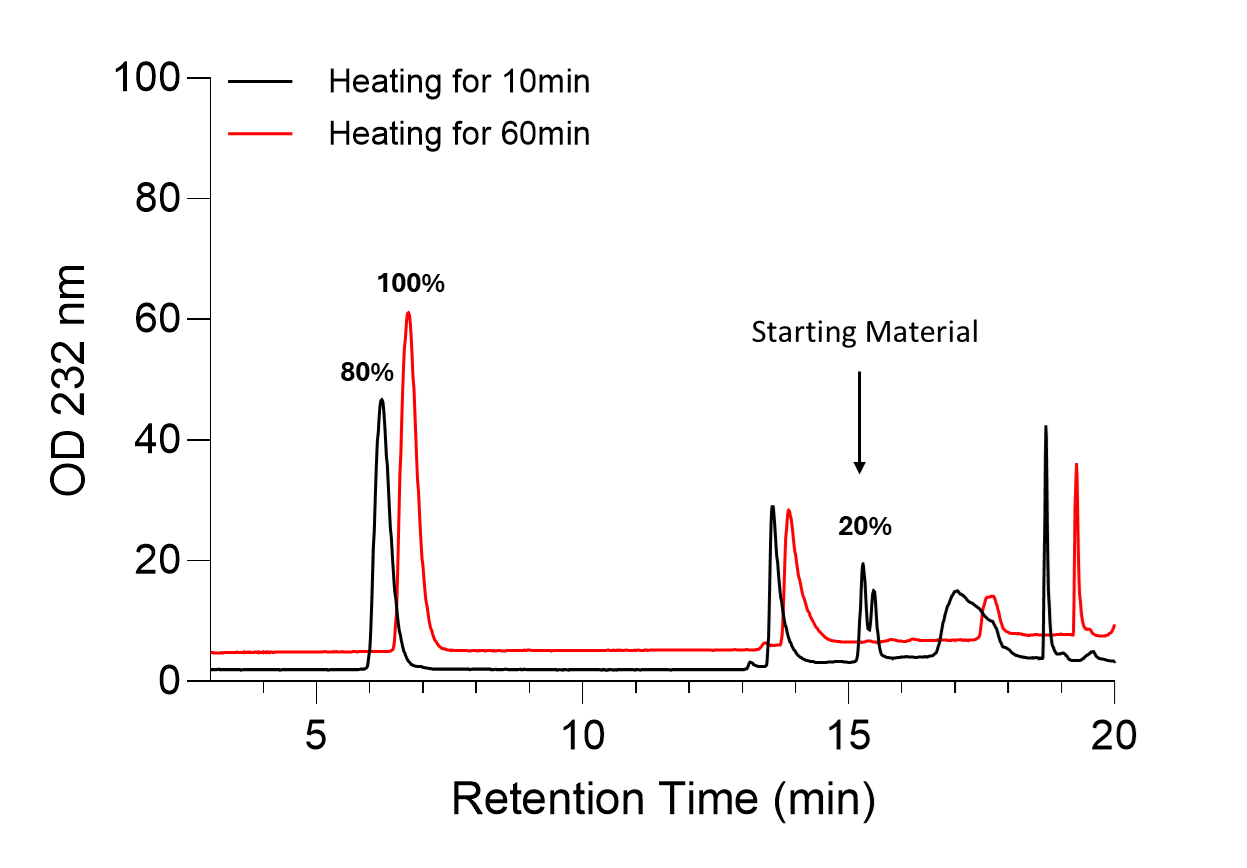


**Supplementary Figure S3. HPLC analysis of HA disaccharide degradation after treatment at 95 °C.** Strong anion-exchange chromatograms of HA disaccharide (eluting at 15.4 min) after 10 min (black) or 60 min (red) at 95 °C in enzymatic digestion buffer (50 mM NaAcO pH 6.0, 1 mM Ca(AcO)_2_, 0.1 g/L BSA). The level of the major degradation product of the disaccharide (eluting at 6.2 min) reached ~80% after 10 min and ~100% after 60 min.


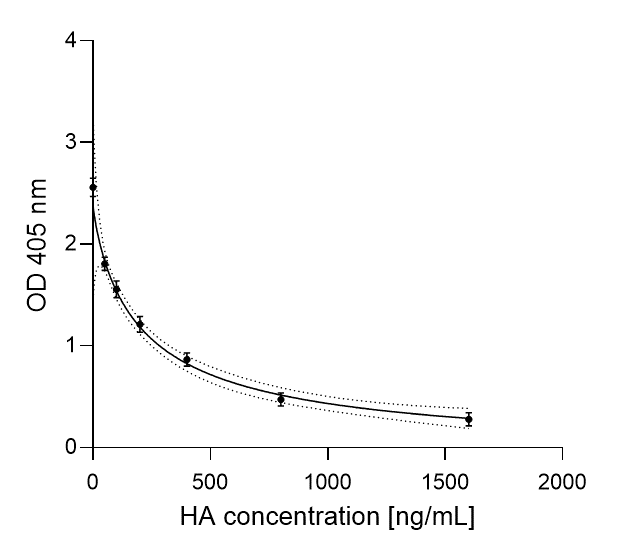


**Supplementary Figure S4. Standard Curve of ELISA kit standard.** Standard curve extrapolated from the OD absorbance at 405 nm of different HA standard concentrations (R^2^ = 0.983). The curve was then used to calculate the concentration of the samples used in this study.

|  |  |  |  |  |  |  |  |  |  |  |
| --- | --- | --- | --- | --- | --- | --- | --- | --- | --- | --- |
|  | | MRM Transition | | | | | | | |  |
| Disaccharide | | Retention Time (min) | | Precursor | Product | | Collision Energy (V) | | Tube Lens (V) |  |
|  | |  |  | | |  | | |  |  |
| HA  ^13^C HA | | 10.45 | | 572.22 | 396.02 | | | 24.67 | 147 |  |
|  |  | 10.45 | | 578.2 | 396.02 | | | 24.67 | 147 |  |

**Supplementary Table S1.** MRM transitions for AMAC labeled native and ^13^C-labeled disaccharide.

**Supplementary Table S2. LC-MS/MS data to estimate HA polysaccharide concentration.** The concentration of different size of HA polysaccharides (HA 33-K, HA 210-K and HA 540-K) was estimated performing LC-MS/MS analysis with internal ^12^C commercial lyase-calibrant. In total 3 measurements per sample were performed to illustrate the robustness of the method. The average concentration is calculated using all the values from each polysaccharide (n=3).

|  | HA  disaccharide ^12^C calibrant AUC | | μg | HA  disaccharide ^13^C  AUC | | μg | | Avg mg/mL |  |
| --- | --- | --- | --- | --- | --- | --- | --- | --- | --- |
| HA 33-K |  |  | | |  | | |  |  |
|  | 10034 | | 0.05 | 295171 | | 1.47 | |  |  |
|  | 7989 | |  | 249521 | | 1.56 | |  |  |
|  | 11669 | |  | 348749 | | 1.49 | | 1.51 |  |
| HA 210-K |  |  | | |  | |  | |  |
|  | 11636 | | 0.05 | 308577 | | 1.326 | |  |  |
|  | 13016 | |  | 345032 | | 1.325 | |  |  |
|  | 12972 | |  | 329095 | | 1.268 | | 1.31 |  |
| HA 540-K |  |  | | |  | |  | |  |
|  | 11253 | | 0.05 | 304329 | | 1.352 | |  |  |
|  | 11547 | |  | 314631 | | 1.362 | |  |  |
|  | 10961 | |  | 317602 | | 1.449 | | 1.39 |  |

**Supplementary Table S3. Total HA amount from mice tissues.** HA content was measured using LC-MS/MS method with ^13^C-HA-disaccharide calibrants. Total HA amount is individually reported for each tissue or plasma (*ng/mL).

| Tissue | HA content (ng/mg) | | | | | Avg. ng/mg | St.Dev. |
| --- | --- | --- | --- | --- | --- | --- | --- |
| Brain | 14.2 | 13.2 | 16.8 | 10.4 | 11.1 | 13.1 | 2.55 |
| Heart | 11.6 | 9.4 | 24.3 | 19.9 | 21.0 | 17.2 | 6.40 |
| Kidneys | 0.75 | 1.0 | 1.56 | 0.61 | 0.77 | 0.94 | 0.37 |
| Liver | 0.23 | 0.31 | 0.41 | 0.19 | 0.22 | 0.27 | 0.09 |
| APAP-Liver | 11.2 | 17.8 | 9.83 | 32.3 | 26.7 | 19.6 | 9.8 |
| Lungs | 2.17 | 3.14 | 7.89 | 2.08 | 2.91 | 3.64 | 2.4 |
| Plasma* | 212 | 133 | 222 | 200 | 86.8 | 171 | 58.5 |
| APAP-Plasma* | <0.1 | <0.1 | <0.1 | 31.7 | <0.1 | 6.33 | 14.2 |
